# An arthroscopic repair technique for proximal anterior cruciate tears in children to restore active function and avoid growth disturbances

**DOI:** 10.1007/s00167-020-06367-w

**Published:** 2021-01-02

**Authors:** Marco Turati, Luca Rigamonti, Nicolò Zanchi, Massimiliano Piatti, Diego Gaddi, Massimo Gorla, Robert J. Omeljaniuk, Aurelien Courvoisier, Marco Bigoni

**Affiliations:** 1 School of Medicine and Surgery, University of Milano-Bicocca, Monza, Italy; 2 Orthopedic Department, San Gerardo Hospital, University of Milano-Bicocca, Via Pergolesi 33, 20900 Monza, Italy; 3 Transalpine Center of Pediatric Sports Medicine and Surgery, University of Milano-Bicocca - Hospital Couple Enfant, Monza (Italy), Grenoble, France; 4 Department of Paediatric Orthopaedic Surgery, Hospital Couple Enfant, Grenoble Alpes University, Grenoble, France; 5 Department of Biology, Lakehead University, Thunder Bay, ON P7B5E1 Canada

**Keywords:** Anterior cruciate ligament, Arthroscopic fixation, Open physes, Paediatric

## Abstract

**Purpose:**

The aim of this study was to assess midterm clinical outcomes in Tanner 1–2 patients with proximal anterior cruciate ligament (ACL) tears following arthroscopic-surgical repair using an absorbable or an all-suture anchor.

**Methods:**

Fourteen (9.2 ± 2.9 years-old) of 19 skeletally immature patients reached the 2 years of clinical follow-up. Physical examinations included the Lachman test, Pivot-shift test, One-leg Hop test, Pedi-IKDC as well as Lysholm and Tegner activity scores; knee stability was measured with a KT-1000 arthrometer. Overall re-rupture rates were also evaluated in all operated patients.

**Results:**

At 2 years post-surgery, the Lysholm score was 93.6 ± 4.3 points, and the Pedi-IKDC score was 95.7 ± 0.1. All patients returned to the same sport activity level as prior to ACL lesion within 8.5 ± 2.9 months, with one exception who reported a one-point reduction in their Tegner Activity score. No leg-length discrepancies or malalignments were observed. Four patients presented grade 1 Lachman scores, and of these, three presented grade 1 (glide) score at Pivot-shift; clinical stability tests were negative for all other patients. Anterior tibial shift showed a mean side-to-side difference of 2.2 mm (range 1–3 mm). The One-leg Hop test showed lower limb symmetry (99.9% ± 9.5) with the contralateral side. Overall, 4 out of 19 patients presented a re-rupture of the ACL with a median time between surgery and re-rupture of 3.9 years (range 1–7).

**Conclusion:**

This surgical technique efficiently repairs proximal ACL tears, leading to a restoration of knee stability and a quick return to an active lifestyle, avoiding growth plate disruption.

**Level of evidence:**

IV.

## Introduction

Participation of paediatric and adolescent children in high-energy sports and associated intensive physical training programs has increased dramatically in recent years. As a consequence, the increased incidence of sports-related injuries, in particular anterior cruciate ligament (ACL) injuries, has increased the need for surgical treatment of these skeletally immature patients [2, 29, 47]. Historically, the treatment of choice for these injuries was conservative; however, recent studies have shown that delayed surgery is correlated with an increased risk of joint instability, medial meniscal tear, and significantly longer time to return to sporting activities [17, 19, 31]. An important consideration is the proximity of the epiphyseal growth plate and the perichondral ring to the femoral ACL footprint. It is well known that skeletal immaturity predisposes patients to risks of growth disturbances [7, 13, 30]. To select the optimal treatment procedure, a pre-operative measurement of skeletal age and Tanner staging, as well as a standing full-length lower limb radiograph is required to highlight any length discrepancies and malalignments [1].

There are different options for ACL reconstruction, and the Tanner stage has a key role in selection of a surgical technique. Among patients with stage 1 or 2, the most common techniques are: (1) extra-physeal iliotibial band (ITB) reconstruction, (2) all-epiphyseal reconstruction, (3) trans-physeal reconstruction with a soft graft, and (4) partial trans-physeal reconstruction [5, 13, 14, 25, 39]. The rate of growth disturbance after ACL reconstruction in Tanner 1 and 2 patients is still a risk and varies from 2 to 13% [9, 15, 27, 40, 49].

Some previous studies demonstrated that ligament tissue of these patients is characterized by greater cell density and greater cellular migration potential [4, 35]. Consequently, there has been a recent increase in ligament repair surgeries for paediatric patients [12].

Historically, surgical repair of ACL tears in young and adult patients was a common open surgical technique; however, it was characterized by excessive failure rates. A careful analysis of specific anatomical details and the timing of intervention revealed that proximal tears and early surgical intervention were correlated with positive outcomes [32, 37, 41, 44].

Moreover, a critical advantage associated with conservation of the native ligament is preservation of proprioceptors, necessary for restoration of proprioceptive capability [37, 41].

Consequently, there has been a recent increase in the number of ACL surgical repairs together with the development of new and diverse repair techniques [3, 11, 33, 43]. DiFelice et al. presented encouraging results on ACL repairs in adults with proximal tears; as well, they introduced the technique of adding supplementary internal bracing [23]. More recently, Bigoni et al. described a novel arthroscopic surgical repair of proximal ACL lesions in paediatric patients [3]. Preliminary results of this repair technique were described; however, midterm outcomes are necessary to assess the joint status and confirm the validity of this technique.

The aim of this study was to assess midterm clinical outcomes in skeletally immature patients with proximal ACL tears who underwent arthroscopic ACL femoral reinsertion with anchor, and represents, to the best of our knowledge, the first such investigation. Good knee stability, optimal patient-reported outcomes and the absence of growth disturbances were expected. This article described also any traumatic ACL re-ruptures and post-operative chondral or meniscal tears observed after paediatric arthroscopic ACL femoral reinsertion.

## Materials and methods

This study was approved by the Institutional Review Board (IRB) of San Gerardo University Hospital (act n. 359, November 13th, 2014). Informed and written consent was collected from every patient; assent of both parents was confirmed in writing in agreement with our protocol previously approved by the local ethics committee and conforming to the principles outlined in the World Medical Association Declaration of Helsinki.

This retrospective case series describes the midterm outcomes of patients who underwent ACL surgical repair with femoral reinsertion. The first objective is to evaluate the midterm knee function, the rate and level of return to sport after surgical ACL repair. The second objective is to describe the rate of ACL reinjury and registered any chondral or meniscal lesions that have occurred during all the follow-up.

For the first aim, every patient was assessed with subjective and objective evaluations during the visit that was performed for each patient 2 years after surgery. Patient-reported outcome measures (PROMs), validated for paediatric patients, included the (1) Pedi-International Knee Documentation Committee (IKDC), and the (2) Lysholm score to measure their clinical state and reveal any symptoms, as well as (3) the Tegner score to assess their sport activity level. During the clinical evaluation, knee stability was assessed using the KT-1000™ knee arthrometer and growth disturbances were excluded by evaluating limb alignment and length.

To detect every reinjury, we encouraged every patient to communicate every trauma and discomfort that they might have experienced during the follow-up. All occurrences were documented and investigated, and diagnoses were confirmed by MRI or diagnostic arthroscopy.

The clinical diagnosis of ACL lesion was followed by radiographic imaging to assess the possible presence of a fracture or spine avulsion, and then by magnetic resonance imaging (MRI) to confirm the diagnosis and verify the location of the ACL tear.

Skeletally immature patients with a proximal ACL lesion (visual and palpatory assessment) and adequate quality of ACL stump were treated with arthroscopic ACL reinsertion using an anchor (absorbable or all-suture) within 6 months of injury. Patients with (1) poor or not acceptable ACL stump quality, or (2) mid-substance or distal ACL lesion arthroscopically diagnosed, or (3) multi-ligament knee injury, did not undergo ligament reinsertion but rather were treated with ACL reconstruction using the ITB autograft technique (modified McIntosh technique) [25].

**Fig. 1:**
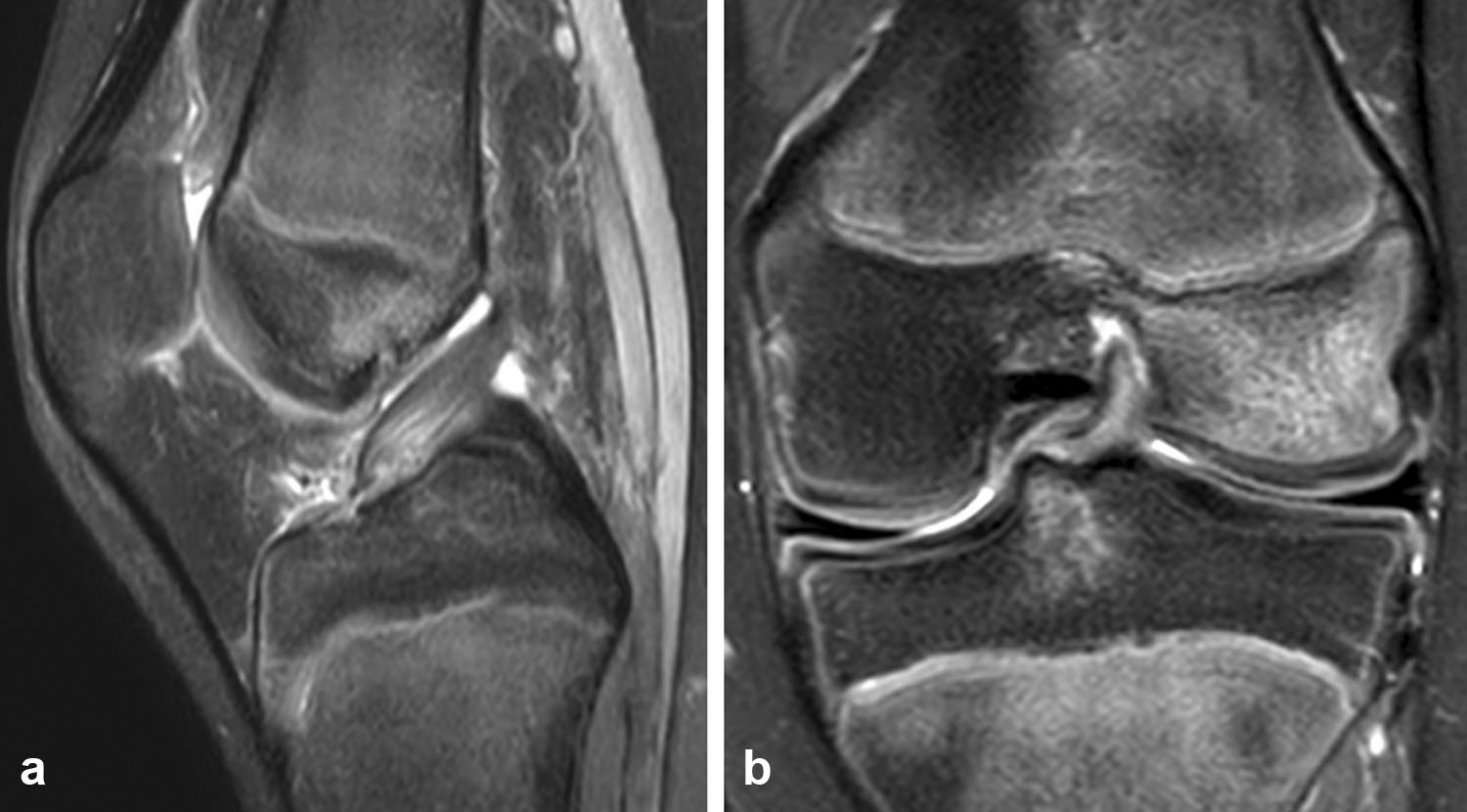
PDW SPAIR (Proton Density Weighted Spectral Attenuated Inversion Recovery) sagittal (**a**) and coronal (**b**) magnetic resonance image of a left knee with a proximal avulsion anterior cruciate ligament tear in a 13-years-old with open growth plates

**Fig. 2:**
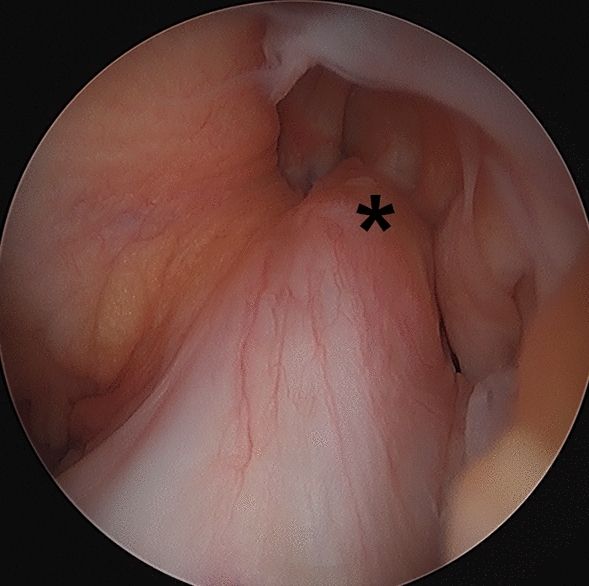
Initial arthroscopic image of a left knee, viewed from the anterolateral portal, with a proximal avulsion tear in a 12-year patient. The ACL stump (asterisk) is avulsed from its insertion at the footprint on the lateral femoral condyle

Inclusion criteria to clinical midterm follow-up were (1) primary ACL proximal lesion clinically diagnosed and confirmed by MRI (Fig. 1), (2) open growth physis confirmed by radiograph, (3) Tanner stage 1–2 confirmed at the time of ACL surgery (assessed after physical examination), (4) intraoperative confirmation of a good quality of ACL stump and location of lesion, and (5) at least 2 years of follow-up. Exclusion criteria included (1) any pre-surgical growth plate disturbances, (2) any previous or new surgical intervention on the same knee side and, and (3) less than 2 years of follow-up.

To evaluate ACL reinjury and any new chondral or meniscal lesions, all the patients underwent ACL surgical repair with femoral reinsertion were considered, also those with less than 2 years of follow up.

### Surgical technique

All surgeries started with a standard arthroscopy of the affected knee joint. Any concomitant chondral and meniscal injuries were treated first. The location of the proximal ligament tear as well as the quality of the stump was evaluated by probing under direct vision (Fig. 2). If arthroscopic examination confirmed the requisite criteria, an accessory trans-patellar tendon portal was created [8].

The proximal extremity of the ACL stump was transfixed using a spinal needle (18-gauge, 3.50 in) through the anteromedial portal; a polydioxanone (PDS) 2–0 (Ethicon, Sommerville, NJ, USA) was passed into the ACL stump through the needle. With the knee flexed at 90°, the ACL stump was re-approximated to its femoral insertion site using a probe to evaluate the feasibility of an anatomical reinsertion. After the femoral footprint was cleaned, the bone just next to the femoral ACL insertion site was drilled with three or four micro-holes to promote bleeding. Next, with the knee flexed at 90°, an appropriate hole was drilled into the origin of the ACL footprint under direct visualization to avoid the femoral growth plate. A curved drill guide (Iconix System, Stryker, CA, USA) was used to achieve the appropriate drill trajectory. From the antero-medial portal, a bio-absorbable or all-suture anchor (Panoloc 3.5 mm, DePuyMitek, MA, USA or Iconix 1.4 mm, Stryker, CA, USA) was inserted into the femoral ACL footprint. Anchor tension and stiffness were tested. Using the PDS 2–0, previously prepared as a shuttle relay, one of the anchor’s sutures was passed through the ACL and the ACL was re-approximated to the original position, tightened with a single sliding knot with self-locking loop (Samsung Medical Center knot [24]) and fixed with two simple knots. The ACL tension and stiffness were tested with a probe.

A custom-made long-leg splint was applied and maintained 4 weeks post-operatively.

### Rehabilitation

Post-operative care consisted of partial weight bearing for 4 weeks and isometric quadriceps activation in extension with the knee maintained in the splint to protect the ACL suture.

After 4 weeks, the long-leg splint was removed followed by rehabilitation therapy. During the first day, patients performed hydro-physiotherapy to promote recovery of muscular control of the limb and a correct gait, followed by a specific muscular training program to improve proprioception and increase the strength of quadriceps and hamstring muscles. After 3–4 months following surgery, when clinical tests and muscular strength values were ascertained, patients started straight-line jogging, open chain and plyometric exercises. Contact Sports were allowed 7–12 months after the surgery.

### Scores and objective outcomes

Pedi-IKDC is specific for knee function measurement in children patients, the score ranges from 0 to 100 where 0 indicates poor function and 95–100 indicates excellent function, with an acceptable test–retest reliability (ICC = 0.91, 95% CI 0.86–0.95) and internal consistency (Cronbach alpha = 0.91) [6, 28, 38]. Lysholm survey was demonstrated to be an adequate instrument to evaluate the functional status of the knee in young patients (adequate validity and responsiveness in paediatric patients; effect size = 1.41; standardize response mean = 1.62), the maximum score of 100 represents optimal knee function [18]. The Tegner Activity scale grades the activity level though an evaluation of work and sport practice, the higher score is related to more intense activity, it is both reliable (ICC = 0.82, 95% CI 0.66–0.89) and consistent (Cronbach alpha = 0.72) [6]. Although this instrument was originally developed for use in adult population, children can provide a precise assessment of their daily activity level [45].

Clinical evaluation included the (1) Lachman test, (2) Pivot-shift test and (3) One-leg Hop for distance test; Lachman and Pivot-shift tests were assessed according to IKDC grade. During the One-leg Hop test, subjects were instructed to jump as far as possible with a controlled leading. Tests were performed with the nonsurgical limb first and then the surgical limb. According to previous studies, a tape measure and stopwatch were used to perform the test [50]. The mean distance of the three trials was used for analysis. Limb symmetry index (LSI) was calculated as the (surgical limb hop distance/non-surgical limb hop distance) × 100.

Possible growth disturbances were assessed by clinical evaluation of limb alignment (with manual goniometer) and length (with tape measure by measuring the distance between the anterior superior iliac spine and the medial malleolus) [42].

Stability of the injured knee was measured using a KT-1000™ knee arthrometer estimating side-to-side differences (SSD, mm) by applying 134 N of force (MEDmetric Corporation, San Diego, California). Anterior knee translation was measured at 30° of knee flexion in both knees; the SSD in anterior tibial translation was calculated by subtracting the value of the contralateral knee from that of the injured knee.

### Statistical analysis

With 14 eligible patients, we have 85% power to detect the complete sport activity recovery (post-surgery minus pre-surgery within 1) for the Tegner score, assuming a standard deviation of 1.1 and a 2-sided significance level of 0.05 (paired *t* test using nQuery Advisor 7.0).

Descriptive statistics were obtained using JMP software package (SAS institute, Cary, NC; version 14). Continuous variables were described as mean ± standard deviation; time and side-to-side anterior tibial shift difference were described as mean and minimum–maximum value range, time between surgery and re-rupture (non-parametric) and Tegner score were described as median and minimum–maximum value range. Difference between pre- and post-operative Tegner score was evaluated with paired *t* test.

## Results

### Patient characteristics

Between 2007 and 2019, inclusive, 20 paediatric patients underwent this arthroscopic surgical ACL repair technique. For the first objective of the study, five of them did not meet the inclusion criteria (four had insufficient follow-up and one presented an ACL re-rupture at 8 months post-surgery consequent to recreational activities), while one patient was lost to follow-up.

Fourteen patients were included in the clinical outcome analysis, 11 male and 3 females. All patients had open physis pre-operatively. The mean age at the time of surgery was 9.2 ± 2.9 years and the mean Tanner score was 1.8 ± 0.6. The mean time between trauma and surgery was 2.4 ± 1.6 months. The ACL repair was performed in 12 patients applying an absorbable anchor (Panoloc 3.5 mm, DePuyMutej, MA, USA), in 2 patients, an all-suture anchor was used (Iconix 1.4 mm, Styker, CA, USA). Characteristics of the patient population enrolled are summarized in Table 1.

**Table 1:** Patient demographics, pre-operative clinical characteristics, and clinical outcomes

	Result
Patient demographics	
Age (years), mean ± SD	9.2 ± 2.9
Male, *n* (%)	11 (79%)
Female, *n* (%)	3 (21%)
Clinical characteristics	
Left injury side, *n* (%)	6 (43%)
Right injury side, *n* (%)	8 (57%)
Contact trauma mechanism, *n* (%)	4 (29%)
Non-contact trauma mechanism, *n* (%)	10 (71%)
Time to surgery (months), mean ± SD	2.4 ± 1.6
Clinical outcomes	
Lysholm score (%), mean ± SD	93.5 ± 4.3
Pedi-IKDC score (%), mean ± SD	95.7 ± 0.1
Tegner activity scale level	
Pre-operative, median (range)	5.5 (3–7)
Post-operative, median (range)	5.5 (3–7)

### Clinical outcomes

At 2 years post-surgery, the mean Lysholm score was 93.5 points ± 4.3, and the mean Pedi-IKDC score was 95.7 ± 0.1. All patients returned to the same sport activity level as prior to ACL lesion, with one exception who reportd a one-point reduction in its Tegner Activity score, the median pre- and post-operative scores were, respectively, 5.5 (range 3–7) and 5.5 (range 3–7) (n.s.). The median time of return to sport activities after surgery was 7.7 months (range 7–8).

Clinical evaluations did not reveal any leg-length discrepancies or malalignment. Four patients presented grade 1 Lachman scores, and of these, three presented grade 1 (glide) score at Pivot-shift; clinical stability tests were negative for all other patients. Anterior tibial shift was evaluated with a KT-1000™ knee arthrometer and showed a mean side-to-side difference of 2.2 mm (range 1–3 mm). Post-operative functional recovery was measured with the One-leg Hop test, the LSI was 99.9% ± 9.5.

### ACL re-ruptures and meniscal or chondral lesions

Nineteen patients who underwent the ACL repair surgery are still in follow-up with a mean time of 5.7 years (range 8–144 months), 4 out of 19 experienced a reinjury (21.1%). The median time between surgery and re-rupture was 3.9 years (range 8–93 months). One patient sustained the reinjury 8 months after surgery, the other three patients reported a complete recovery of their sport activities, did not experience any previous knee instability and reported an new trauma during sport activity as the cause of the knee sprain. About the second surgery, one patient underwent an extra-articular ACL reconstruction with the modified McIntosh Technique using the iliotibial band, three patients sustained an ACL reconstruction with transtibial technique and bone–patellar–bone graft.

Overall, only 1 out of 19 subjects received a meniscal repair for a posterior lateral meniscal tear concomitant to the ACL reinjury, an arthroscopic FasT-Fix device was used for the meniscal repair (Smith & Nephew Endoscopy, Andover, MA, USA). No other chondral or meniscal lesions were registered during the follow-up among all the patients who underwent the ACL repair surgery.

## Discussion

The most important finding of the present study was that the ACL repair technique used in this group of skeletally immature patients was safe and yielded optimal midterm outcomes.

The first aim of this study was to clarify the midterm outcomes of ACL repairs in paediatric patients. At 2 years after surgery, both subjective and objective evaluations showed very good results, with restoration of knee function to pre-injury levels, good joint stability, and optimal symptomatic scores and resumption of daily activities. Almost all the patients reported a return to sport at the same pre-injury level with a time between surgery and recovery of activity of about 8 months on average; only one patient returned to sport at a lower level.

These data are consistent with the preliminary outcomes in the same group of patients, so we confirm that short-term outcomes were maintained also at 2 years following surgical ACL reinsertion [3]. These good midterm outcomes after ACL repair are confirmed in both paediatric and adult studies with optimal subjective scores, restoration of knee stability and high return to sport rate [10, 34, 43]. Gagliardi et al. as well, found that adolescent patients treated with an ACL repair and who did not have a re-injury, had PROMs comparable to ACL reconstructed patients [16].

The re-injury rate in our study was 21.1% (4/19 patients) and a mean time after repair of 4.1 years. There are very few documented cases of ACL repair in paediatric and adolescent patients. A small case series reported no re-ruptures using an augmentation device during the ligament suture [43, 48]. By contrast, another study with a larger patient population described a higher re-injury rate of 48.8% in the repair with augmentation, compared to a 4.7% failure rate of ACL reconstructions at 3 years after surgery [16]. That case–control study was retrospective, without subject randomization and presented some group’s differences as younger age at surgery and a higher percentage of skeletally immature subjects in the repair group. Differences in post-operative protocols, surgical techniques and patient’s characteristics may explain the lower re-injury rate found in our study.

It is important to consider that the re-injury rate is higher in skeletally immature patients who undergo ACL reconstruction compared to adults, and in this series, the mean age at surgery is low with 9.2 years. Webster et al. reported a re-rupture rate of 18% and contralateral ACL lesions of 17.7% in a group of 316 adolescents with a mean age of 17.2 years-old who underwent trans-physeal ACL reconstruction with hamstring tendon graft [46]. In another large study of 561 patients under 20 years-old, a re-injury rate of 14.5% was reported after a different type of ligament reconstruction (trans-physeal with hamstring graft, all-epiphyseal and ileo-tibial band). Recently, Kocher et al. reported a lower re-rupture rate in paediatric ACL reconstruction with iliotibial band autograft. They reported a re-injury rate of 6.6% in a group of 137 knees, but the average reported follow-up time was 33.5 months, so it is important to verify if this rate will be maintained also at long term [26].

The present study has some limitations including a small subject cohort, the retrospective nature of the research, and the absence of a control group (as a group of patients of the same age and skeletal maturity that undergo ACL reconstruction) [12].

Other limitations of this case series are the high dropout and re-injury rate after ACL repair compared to other reconstruction techniques performed in skeletally immature patients. However, it is also useful to notice that these re-injuries occurred at a mean time of 4.1 years post-surgery and almost all the patients had resumed their previous levels of sport activity and described their knee as normal as before the re-rupture. More significantly, the rate of new chondral or meniscal lesion registered was very low (1/19). Nevertheless, the rate of reinjury remains a criticism of this technique, future studies with larger number of subjects and longer follow-up are needed to better understand its utility and safety.

As well, the absence of transfixing fixation without bone tunnels or internal bracing may not permit an objective tensioning of the repaired ACL [16]. Our technique restores ACL continuity and supports intrinsic healing capability in these selected patients, future studies should evaluate mechanical properties of repaired ACL.

Time between injury and surgical repair may influence ACL stump tissue quality; for this reason, we try to minimize the time to surgery. Future studies should evaluate how time between injury and surgery influences ligament tissue quality.

The ACL repair technique proposed here has some advantages including (1) no risk of damage to growing cartilage, (2) use of only an absorbable or all-suture anchor without any other foreign material that could necessitate subsequent revision surgery, and (3) no graft donor site comorbidities. Moreover, re-injured patients were approaching skeletal maturity thereby enabling selection of the best treatment, usually adult reconstructive surgery.

Another possible advantage is that by preserving native ligament tissue, proprioceptive structures, like mechanoreceptors and free nerve branches, can be maintained. It is well known that one of the possible causes of re-injury and contralateral ACL lesions is the lack of proprioception and muscular strength, so adopting a surgical technique that preserves the native tissue and helps in the restoration of the pre-injury proprioception capability would significantly benefit the patient. Future studies should evaluate the potential consequences of the preservation of these neural structures [20, 21, 36].

Ultimately, the goal of this new surgical technique is to improve the type of surgical option to customize the treatment on patient’s characteristics and needs. This ACL repair technique represents an alternative to reconstruction in cases of proximal ligament lesions. Key indications for paediatric ACL repair are repairable lesions, instability after high-quality rehabilitation and unacceptable modification of activity level [1].

During the last years, the use of this ACL repair technique increased in the adult population with encouraging results on subjective outcomes and re-injury rates, greater results could be expected in paediatric patients as they present a higher healing potential, in particular children with Tanner stage 1 and 2, compared to adults [22, 23, 33, 34].

## Conclusion

These findings support the use of this ACL repair technique in patients with a proximal lesion to safely permit the patient to resume sport activities without placing at risk of growth disruption. This option should be considered in clinical practice with due consideration of the type of ACL lesion and growth plate status, both of which are key to guiding the surgical decision.
